# Institutionalizing medical certification of cause of death training: experiences and insights from seven countries

**DOI:** 10.1080/16549716.2026.2732793

**Published:** 2026-09-23

**Authors:** Rodela Anjum Khan, Beatriz Plaza, Fatima Marinho, Nazrul Islam, Moyeen Uddin, Nelson Alí Gordillo Alfonso, Aura Liliana Torres Umbarila, Wubetsh Ayenew, Yeshitila Worabu, Azeb Atraga, Tangut Azeze, Selam Dergeba, Muluken Haile, Hailu Mio, Winfred Nzioka, David Kariuki, Esther Mutheu, Godfrey Ngoboka, Albert Tuyishime, Rangana Wadugedara, Mayakaduwa Nimdinu, Lakshmi Somatunga, Gisbert Msigwa, Claud Kumalija, Grace Magembe, Trust Nyondo, Robert Mswia, James Mwanza, Philip Setel, Romain Santon, Martin Bratschi

**Affiliations:** a Independent Consultant, Portugal; b Public Health Programs, Vital Strategies, New York, NY, USA; c Ministry of Health, Ethiopia; d Kenya Medical Practitioners and Dentists Council, Kenya; e Rwanda Biomedical Centre, Rwanda; f Public Health Services, Government of Sri Lanka Ministry of Health Sri Lanka, Sri Lanka; g Directorate of Monitoring and Evaluation, Ministry of Health, Tanzania, United Republic of Tanzania; h Chief Medical Officer, Ministry of Health, Tanzania, United Republic of Tanzania

**Keywords:** Medical certification cause of death, Civil Registration and Vital Statistics (CRVS), health systems strengthening, medical education, qualitative research

## Abstract

**Background:**

Accurate medical certification of cause of death (MCCD) is indispensable to producing high-quality mortality data for public health planning and health systems. While training is known to improve MCCD quality, countries may struggle to institutionalize training programs sustainably.

**Objective:**

This study examines the approaches taken by seven countries across three world regions – Bangladesh, Colombia, Ethiopia, Kenya, Rwanda, Sri Lanka, and Tanzania – to institutionalize MCCD training within national medical education frameworks.

**Methods:**

This qualitative multi-country study used purposive sampling of countries participating in the Bloomberg Philanthropies Data for Health Initiative. Data were primarily derived from in-depth stakeholder interviews. Thematic analysis identified key enablers, challenges and strategies; all of which were validated through country review and triangulation.

**Results:**

Common enablers of the institutionalization of MCCD training included government commitment, early stakeholder engagement, integration into pre-service and in-service training, and international partnerships. E-learning and training of trainers expanded their reach, while weak coordination constrained implementation. Linking training to licensure renewal or continuing professional development credits increased uptake. Scale-up required regional adaptation in both decentralized and centralized systems. Long-term sustainability requires integration into national budgets and regulatory frameworks.

**Conclusions:**

Institutionalizing MCCD training is a long-term process shaped by political commitment, regulatory alignment, stakeholder coordination, and flexible training delivery. Findings provide practical insights for embedding MCCD training within national systems, including strengthening legal frameworks, leveraging continuing professional development programs, adapting regionally, and planning for domestic financing.

## Paper Context

- **Main findings:** Institutionalizing training on medical certification of cause of death requires sustained government commitment, cross-sector coordination, and integration of training into both medical education and continuing professional development systems.
- **Added knowledge:** Drawing on experiences from seven countries, this study identifies practical strategies and common barriers involved in embedding medical certification of cause of death training into national health systems.
- **Global health impact for policy and action:** Strengthening routine training on medical certification of cause of death can improve the quality of mortality statistics, though translating better data into better decisions also requires political will, institutional capacity for data use, and policy environments in which mortality evidence is proactively addressed rather than deprioritized.

## Background

This paper analyzes the challenges, successes, and strategies related to the institutionalization of Medical Certification of Cause of Death (MCCD) training in seven countries across three world regions: Bangladesh, Colombia, Ethiopia, Kenya, Rwanda, Sri Lanka, and Tanzania. It highlights actionable insights for countries looking to strengthen their national MCCD practices.

A well-functioning civil registration and vital statistics (CRVS) system is responsible for registering births and deaths, issuing birth and death certificates, and compiling related statistics [1]. It also disseminates birth and death data for policymaking, including information on the causes of death (COD). Improving and maintaining MCCD practices within the CRVS system is essential for producing high-quality COD data, coded according to the International Classification of Disease and Related Health Problems (ICD) mortality rules to generate statistics that inform evidence-based population health policies [2,3].

Routine training on MCCD best practices both within clinical education curricula of medical schools (pre-service) and for in-service physicians is crucial to the quality of cause-of-death data produced within a country [4].

As defined by Haghighi and Takian: ‘Institutionalization in health refers to the process of establishing and embedding health policies, programs, and practices within a system, organization, or community.’ It is ‘an ongoing process in which a set of activities becomes an integral and sustainable part of a formal system’ [5]. The authors identify several interdependent dimensions through which health programs become sustainably embedded within systems: political commitment and governance support, multi-stakeholder engagement and coordination, regulatory and legislative alignment, capacity building, resource integration, and monitoring and feedback [5]. This framework has been applied to understand how health policies transition from externally supported initiatives to domestically owned system components – a process particularly relevant in low- and middle-income contexts where donor dependency poses long-term sustainability challenges. We draw on this framework as an interpretive lens to contextualize our findings on MCCD training institutionalization across seven countries, examining how the patterns that emerged from the data align with and extend our understanding of these dimensions across diverse health system contexts. While global guidance discusses the importance of MCCD within CRVS systems, there is limited literature that examines the pathways and processes that different countries take to institutionalize MCCD training at a national level [6].

Institutionalizing MCCD training improves the accuracy and reliability of mortality statistics, strengthens CRVS systems, and supports better population health and health system decision-making. Correct completion of the MCCD form counts on the certifying doctor following ICD guidelines, while other stakeholders can ensure that the data extracted from the MCCD are accurately recorded and used to produce vital statistics [7]. However, studies have shown that errors in medical certification of the cause of death are prevalent globally. This may result from minimal physician training, inadequate feedback or audit systems, or clinical urgencies that prevent physicians from prioritizing MCCD [2,8]. Unreliable cause-of-death data affect public health statistics, may mislead policy decisions, and result in misguided health interventions [9–12].

Successful institutionalization at the national level requires a multifaceted approach addressing both new physicians and in-service clinicians: integrating MCCD training into medical curricula (pre-service), providing continuing professional development (in-service), and establishing auditing systems to monitor compliance and accuracy [8].

Because of the lack of formal training on MCCD during pre-service medical education, particularly for interns and junior doctors, there are significant knowledge gaps in MCCD practices [13]. For in-service doctors, continuing professional development (CPD) serves as a critical tool for health professionals to maintain, enhance, and broaden their knowledge, skills, and performance, ultimately contributing to improved quality of care delivery [14]. Studies have demonstrated that well-structured CPD programs – especially using mandatory and interactive approaches – can positively affect health professional behavior and competencies [15,16].

Training interventions have been shown to improve the quality of MCCD significantly. A systematic review indicated that structured training programs enhance the knowledge and skills of healthcare providers, leading to a reduction in errors in medical certification of cause of death [17]. In Tanzania, the introduction of a comprehensive training package resulted in improved MCCD quality and subsequently better mortality statistics within 1 year of implementation [18]. Despite its importance, many countries face challenges in institutionalizing MCCD training, including the lack of a standardized curriculum, insufficient political commitment, and resource constraints.

This article is intended for professionals in medical education, public health statistics, health informatics, and health system decision-making, particularly CRVS stakeholders and policymakers seeking to strengthen MCCD practices. Drawing on qualitative interviews, it highlights lessons on government commitment, training strategies, and stakeholder engagement rather than describing each country’s institutionalization process in detail.

## Methods

This study employed a qualitative approach to explore contextual processes, stakeholder experiences, and institutional dynamics that are not adequately captured through quantitative methods. Countries were purposively selected from those participating in the Bloomberg Philanthropies Data for Health Initiative [19]. Seventeen countries were contacted by email with a study concept note, and eligibility was determined based on the country teams’ responses to the following questions:

- *Is there a formal order from the relevant CRVS authority (or authorities) in your country mandating the inclusion of MCCD in medical education curricula (pre-service)?*
- *Is there a formal order from the relevant CRVS authority (or authorities) in your country mandating the inclusion of MCCD in continuous medical education (in-service)?*

Seven countries that responded affirmatively to at least one question were included in the study: Bangladesh, Colombia, Ethiopia, Kenya, Rwanda, Sri Lanka, and Tanzania. These countries represent a diverse range of health system structures and varying levels of MCCD training policies. Participants were country team members or partners of the Bloomberg Philanthropies Data for Health Initiative, including ministry of health officials, health regulatory staff, university deans, and representatives from international organizations such as WHO and Vital Strategies. These stakeholders were directly involved in the design, implementation, and evaluation of MCCD training in their countries.

The study protocol was reviewed by the BRANY Institutional Review Board (USA), determined to be exempt from full review, and conducted in accordance with the ethical principles of the Declaration of Helsinki. Verbal informed consent was obtained from all participants because interviews were conducted virtually and the study posed minimal risk, making written consent unnecessary. Participation was voluntary, and participants could decline to answer questions or withdraw at any time. Findings were reported by country without identifying individual participants, and interview summaries were shared with country partners for validation.

Data collection took place in 2024 through virtual semi-structured interviews (Supplement A) covering key issues related to the institutionalization of MCCD training. Interviews were conducted by researchers with professional experience in global health systems strengthening and CRVS initiatives and were audio-recorded with participants’ approval for transcription. Study materials were securely stored in accordance with the institutional data management procedures. Given the small number of country-level informants and the contextual nature of the discussions, anonymized interview data are not publicly available because of the potential risk of indirect identification.

The research team included technical assistance partners involved in the design and implementation of MCCD training programs in all study countries. This provided valuable contextual knowledge and facilitated candid discussions of implementation challenges but also introduced the potential for bias. To address this, findings were triangulated with independent documentary evidence, interview summaries were validated by country partners, and interpretations were discussed among researchers with varying levels of country involvement. Findings should therefore be interpreted with anawareness of the research team’s role.

## Results

Data were analyzed using inductive thematic analysis, with themes derived from the data rather than predetermined [20]. Interview transcripts and summaries were reviewed iteratively to develop codes from recurring patterns across countries. Emergent themes were then interpreted using Haghighi and Takian’s health institutionalization framework [5] to examine how MCCD training became embedded within national systems. Findings were compared across countries, and credibility was strengthened through triangulation with national policy documents and legislation, validation of interview summaries by country partners, and ongoing analytic discussions among researchers to enhance reflexivity and consistency. Table 1 summarizes the participant characteristics and country partners consulted for triangulation.

**Table 1.** Participant overview.

Country	Participants	Role and Organization	Data Triangulation
Bangladesh	2	Country Coordinators, Bloomberg Philanthropies Data for Health Initiative, Vital Strategies	Ministry of Health and Family Welfare Directorate General of Health Services (DGHS), Bangladesh Medical and Dental Council (BMDC), and the Health Education Bureau (HEB)
Colombia	1	Country Coordinator, Bloomberg Philanthropies Data for Health Initiative, Vital Strategies	Ministry of Health and Social Protection, National Institute Legal Medicine and Forensic Sciences
Ethiopia	2	Country Coordinators, Bloomberg Philanthropies Data for Health Initiative, Vital Strategies	Ministry of Health, Ethiopian Medical Association
Kenya	1	Country Coordinator, Bloomberg Philanthropies Data for Health Initiative, Vital Strategies	Ministry of Health, Kenya Medical Practitioners and Dentists Council
Rwanda	1	Country Coordinator, Bloomberg Philanthropies Data for Health Initiative, Vital Strategies	Ministry of Health, Rwanda Biomedical Centre, Rwanda Medical and Dental Council
Sri Lanka	1	Country Coordinator, Bloomberg Philanthropies Data for Health Initiative, Vital Strategies	Ministry of Health, Sri Lanka, Office of the Additional Secretary (Public Health Services)
Tanzania	1	Country Coordinator, Bloomberg Philanthropies Data for Health Initiative, Vital Strategies	Ministry of Health, Directorate of Monitoring and Evaluation (HMIS Unit)
Total Participants	**9**		

Five cross-cutting themes emerged from the iterative analysis: government commitment, stakeholder coordination, international partnerships, training integration, and professional development incentives. These themes are discussed below, citing country examples, illustrating both challenges and facilitators that influence the sustainability and reach of MCCD training programs.

### Government commitment and political will

Government buy-in has emerged as an essential factor shaping the success of MCCD training institutionalization across countries. Early and strong government commitment was shown to facilitate the integration of MCCD training at the national level.

In Tanzania, the Ministry of Health led MCCD improvement efforts starting in 2015, supported by the Directorate of Policy and Planning, after acknowledging that mortality data were not aligned with WHO international standards. Similarly, Rwanda’s Ministry of Health took early ownership of the process, and the discussion and implementation of MCCD training began as a part of the National Civil Registration and Vital Statistics Work Plan (2017–2023). With a commitment from the Ministry of Health to develop and focus on strong partnerships at the sub-national, Ethiopia has laid a solid foundation for scaling up its MCCD training program nationwide.

In Kenya, the Kenya Medical Practitioners and Dentists Council spearheaded early efforts in MCCD training, which were then bolstered by broader governmental and county-level support. In Colombia, the Ministry of Health and Social Protection led a proactive strategy on MCCD training, facilitating collaboration across sectors. However, there were barriers due to the lack of a compulsory mandate on MCCD training, which limited its full integration into the medical education curriculum.

Bangladesh and Sri Lanka demonstrated sustained government support for improving mortality data through Ministry of Health-led initiatives linked to broader CRVS strengthening efforts. In Bangladesh, support from the Ministry of Health and Family Welfare facilitated the integration of MCCD training within CRVS activities and supported the broader institutionalization process. In Sri Lanka, initial efforts focused on improving mortality data quality in government hospitals before expanding into routine in-service and pre-service MCCD training for medical officers. In 2022, the Ministry of Health incorporated MCCD into continuous professional development (CPD) requirements. Sri Lanka also piloted a master trainer model across 25 hospitals, with trainers responsible for delivering MCCD training to interns and ranked medical officers at their respective facilities before later expanding the approach to additional hospitals.

### Stakeholder engagement and coordination

Institutionalization requires the involvement of diverse stakeholders, including government agencies such as ministries of health, medical councils, universities, and international organizations [5].

In Tanzania, collaboration with the Tanzania Commission for Universities and the National Council for Technical Education enabled the incorporation of MCCD training into medical curricula across universities and lower-level training institutions. Their regulatory oversight of pre-service medical education created a mechanism for integrating MCCD training into both pre-service and in-service education systems.

In Bangladesh, the Directorate General of Health Services coordinated with medical education institutions to integrate MCCD protocols into national training systems and oversee the implementation of standardized certification practices across healthcare facilities. Similarly, in Sri Lanka, collaboration between the Ministry of Health, medical councils, universities, and international partners supported the integration of MCCD training into both pre-service and in-service curricula.

Sri Lanka also demonstrated coordination across community- and facility-based certification systems. Because a proportion of deaths are certified by general practitioners in community settings, the Ministry of Health worked with the College of General Practitioners to provide in-service MCCD training for General Practitioners. The Ministry also coordinated with the Registrar General’s Department and Ministry of Public Administration to update and formally gazette WHO-aligned MCCD formats for both hospital and community use.

In Ethiopia, early coordination challenges between central authorities and regional actors led to greater engagement with regional stakeholders, particularly in rural areas, to adapt training approaches to local implementation contexts. In another East African country, early Ministry-led efforts to institutionalize MCCD training did not progress due to limited coordination between medical schools, health institutions, and regulatory bodies. Subsequent collaboration between the national medical and dental practitioners body, universities, professional authorities, and international partners created a broader institutional framework for implementation.

Colombia and Rwanda similarly involved universities, government agencies, and international partners in the integration of MCCD training into their national health and medical education systems.

### International partnerships and technical assistance

International organizations and partners provided technical assistance and financial support that helped countries design and scale up MCCD training, standardize materials, and establish learning platforms. In Tanzania, for example, external technical assistance enabled alignment with WHO standards across both pre-service and in-service settings. Ethiopia and Kenya similarly leveraged these partnerships effectively. Although logistical challenges may initially slow implementation – as seen in Sri Lanka and Colombia – external technical assistance can provide valuable proof of concept to support long-term institutionalization.

### Integration of MCCD training into medical curricula: pre-service and in-service

Tanzania, Rwanda and Sri Lanka exemplified the integration of MCCD into pre-service education, enabling students to enter the medical workforce with foundational MCCD knowledge. Tanzania incorporated MCCD training into middle college and university curricula by 2017, collaborating with regulatory bodies like the National Council for Technical Education and the Tanzania Commission for Universities to standardize training. Rwanda partnered with universities to establish MCCD as a core component of medical education by 2020. Sri Lanka has developed a uniform curriculum on MCCD for medical undergraduates, which is being implemented by all the medical faculties in the country. The curriculum was developed with partnership by the departments of Public Health, Forensic Pathology, and Medical Education, under the guidance of the Ministry of Health.

In 2017, the Bangladesh Medical and Dental Council issued a circular officially incorporating an MCCD course module into the medical education curriculum, at the advice of the Bangladesh Ministry of Health. This action was preceded by a pilot MCCD training program for in-service doctors, emphasizing its importance for improving vital statistics in the country. There are 107 medical schools in Bangladesh and while these are affiliated with medical universities, they all follow a uniform curriculum set by the Council, but universities oversee respective academic activities and conduct examinations of medical schools under the Council’s jurisdiction.

In contrast, Colombia faced challenges integrating MCCD due to the autonomy of medical schools, which independently set curricula. This challenge underscores the importance of early collaboration with educational institutions and regulatory bodies for sustainable MCCD curriculum integration.

These findings highlight the importance of regulatory support, standardized curricula, and adaptable in-service training. Where medical schools have curricular autonomy, sustained collaboration with educational institutions and regulators is essential.

For in-service training, flexible modalities such as e-learning and face-to-face workshops have proven effective in addressing immediate knowledge gaps among practicing clinicians. However, achieving sustainable integration requires mandatory participation, strong regulatory oversight, and alignment with licensure and professional development frameworks. A robust training program integrating MCCD into both pre-service (medical education) and in-service (continuing professional development) curricula ensures sustainability.

#### E-learning platforms

E-learning platforms were used across all countries in this study to support the scale-up of MCCD training, particularly where face-to-face training was resource-intensive or difficult to deliver consistently across regions. However, implementation experiences varied considerably depending on internet infrastructure, platform functionality, institutional support, and clinician familiarity with digital learning tools.

Rwanda rapidly deployed a national e-learning platform and reported high course completion rates, supported in part by broad internet coverage and integration with existing professional training systems. In Kenya, the Kenya Medical Practitioners and Dentists Council developed an asynchronous e-learning platform for both pre-service and in-service clinicians, with plans to integrate the course into CPD systems. Tanzania launched an online platform in 2020 to support clinicians unable to attend in-person sessions, although technical issues linking course completion to CPD point tracking slowed uptake in some settings.

Several countries used blended approaches that combine virtual and face-to-face training. In Colombia, online modules hosted through the Escuela Superior de Administración Pública extended training access to rural regions while maintaining institutional ownership within the public university system [21]. In Ethiopia, the in-service MCCD course was integrated into the Ethiopian Medical Association’s CPD e-learning platform. Bangladesh increasingly shifted toward online delivery after early workshop-based models proved logistically difficult to sustain at scale, although internet access and resource limitations remained barriers in some rural areas [22–24].

Sri Lanka relied primarily on face-to-face training, particularly for in-service medical officers, due to infrastructure limitations during the country’s economic crisis and varying levels of digital literacy among clinicians [25]. Although a mobile application and virtual learning approaches were developed, implementation remained limited. Instead, the Ministry of Health distributed approximately 20,000 printed MCCD guidebooks to hospitals nationwide as a standardized training resource.

These experiences suggest that e-learning can expand training reach, but implementation depends heavily on local infrastructure, digital literacy, and integration within existing systems. Where digital infrastructure is constrained, blended or paper-based approaches may remain necessary to ensure equitable access.

#### Training of trainers as a capacity-building strategy

Establishing training-of-trainers (TOT) programs proved essential for ramping up MCCD training practices and allowing broad dissemination. Countries with well-established TOT programs were able to scale MCCD training promptly, with national TOT teams often becoming critical in capacity-building efforts across regions and health facilities. Findings from our study demonstrate the benefits of well-developed TOT models that later enable regional dissemination of MCCD training. Furthermore, cascade training is an efficient approach to address health worker shortages in low- and middle-income countries where specialist capacity is limited [26].

Tanzania’s TOT model was launched in 2016 and provided a foundation for scaling MCCD training nationwide. National trainers and clinicians from teaching hospitals and universities who were trained on medical certification of cause of death according to WHO standards led face-to-face sessions, cascaded training and skills to health facilities, including in remote regions. This was a key step as these hospitals produce a significant number of clinicians who would later practice throughout different regions. Tanzania’s TOT model highlights how investing in a structured national training framework, supported by international collaboration and phased regional implementation, can effectively institutionalize MCCD practices from the outset. In Rwanda, the Ministry of Health implemented a phased training approach to ensure national compliance and capacity-building in MCCD. Initially, master trainers, including clinical service directors from referral and provincial hospitals, were trained in MCCD and ICD-10 coding. These directors then trained their colleagues within their institutions, creating a ripple effect for knowledge dissemination. This comprehensive strategy underscores Rwanda’s commitment to eventually reach MCCD institutionalization.

Sri Lanka’s master trainers were initially trained in Colombo’s main teaching hospitals and were instrumental in scaling MCCD training across the country. The pilot project then expanded to 25 hospitals across nine provinces, using a convenience sampling method, and incorporating the largest hospitals in each province. Approximately 125 master trainers were trained in person, and each hospital developed its own rollout plan for further training within their institution. Bangladesh TOT programs ensured wide coverage particularly by focusing on training at large teaching hospitals. The use of a TOT model allowed Bangladesh to cascade training efficiently, with trained professionals further disseminating MCCD knowledge to healthcare workers across the country. Ethiopia also implemented a TOT approach on training healthcare workers in rural areas. First, a national TOT team composed of government officials, medical doctors, and experts from major hospitals was established. The program was then rolled out, focusing on training medical professionals and building capacity within key institutions such as hospitals and universities. The goal was to expand MCCD training to all regions through a decentralized model. Focus areas included developing region-specific training programs and adapting international best practices to the local context. The Ministry of Health has held multiple national and regional-level training sessions since adopting ICD-11 and revising the MCCD format. Although multiple national and regional trainings have been conducted, in-person delivery remains costly and logistically complex, particularly when clinicians must leave their service posts to attend regional sessions.

Across contexts, structured TOT models, phased regional expansion, and institutional embedding within medical education systems are critical to scaling MCCD training while addressing financial and logistical constraints.

### Incentivizing or mandating MCCD training

Maintaining clinician motivation and ensuring compliance with MCCD certification standards has posed challenges across several countries, particularly where MCCD training remains voluntary. Conversely, mandatory training has been shown to improve adherence significantly. Self-motivation to follow guidelines varies, particularly among in-service clinicians. Medical societies and doctors initially had the tendency to be resistant to MCCD when it was introduced because of the ‘extra’ work for them to complete. To counter this, several country partners held numerous meetings and workshops to secure buy-in from medical societies first, who could then communicate directly and more effectively to physicians about their commitment to MCCD implementation, given their professional authority.

In Colombia, integrating MCCD training into pre-service medical curricula has resulted in better compliance among younger clinicians. However, for in-service doctors, the absence of a mandate and limited incentives has led to low completion rates, with only half of those who start the virtual course finishing it. Discussions to make the training compulsory for license renewal are ongoing but have yet to be implemented. Respondents from Tanzania also reported challenges with inconsistent adherence, as clinicians often perceive MCCD as optional despite the availability of CPD credits for completing its e-learning course. Although not currently mandatory, the course is expected to become a requirement for license renewal in the future. Similarly, in Kenya, challenges with compliance have prompted the Kenya Medical Practitioners and Dentists Council to move toward mandating MCCD training for licensure, with plans to allocate up to 10 CPD credits, making it one of the more highly valued CPD activities.

In Bangladesh, the lack of a formal system for mandatory CPD requirements has hindered widespread adoption of MCCD standards, particularly among clinicians who have not received an introductory MCCD training. To integrate MCCD into CPD effectively, Bangladesh must first establish a comprehensive licensing and CPD framework. Ethiopia faces a similar situation where MCCD training, while part of the CPD program, remains optional. Despite offering 19 CPD credits for its MCCD module, compliance is low, especially in rural areas with limited resources and trained personnel. Discussions about making the training mandatory are ongoing but require additional funding and institutional support.

Rwanda’s centralized governance structure, high national internet penetration, and well-integrated CPD and licensure system created enabling conditions for mandatory MCCD training that may not exist in all settings. Systematic training led by senior clinical directors and the introduction of a mandatory e-learning course mitigated resistance among physicians, who viewed MCCD as extra administrative work. This course, accredited by the Rwanda Medical and Dental Council, is self-paced, evaluated rigorously, and required for annual license renewal. Although MCCD training is integrated into the CPD framework in Sri Lanka, CPD points are not mandatory for license renewal, reducing incentives for consistent participation.

Incentivizing or mandating MCCD training can significantly improve compliance, while voluntary participation often results in inconsistent application. Regular audits, feedback mechanisms, and senior hospital leadership can further encourage ongoing adherence. Also, engagement with medical councils or other professional organizations relevant to physicians can help to emphasize the importance of in-service training on MCCD even in the absence of required credit points. Periodic and planned refresher courses and regional workshops may further sustain clinician interest and compliance [7]. Integrating MCCD training into national CPD frameworks and making it mandatory for licensure renewal can enhance compliance and accountability among clinicians, reflecting positive governance principles that are characterized by transparency and participation [5].

Ethiopia’s need to translate MCCD materials into multiple local languages presents significant cost and logistical challenges. Addressing such disparities requires a thoughtful allocation of resources, ensuring that training materials are contextually relevant and accessible. Prioritizing translation efforts and focusing on underserved areas in both pre-service and in-service training can bridge regional gaps and ensure equitable access to MCCD training. Careful consideration and planning within national and regional contexts are crucial to guarantee that MCCD training is implemented in an equitable manner.

Implications for global MCCD efforts suggest a focus on integrating training into both pre-service and in-service medical education, ensuring sustainability through national budget allocations. We have highlighted the importance of structured, scalable approaches underpinned by political commitment and cross-sector collaboration.

## Discussion

This study examined how MCCD training was institutionalized across seven countries, highlighting the diverse pathways through which the training was embedded within national health systems. The findings align closely with the health institutionalization framework proposed by Haghighi and Takian [5], particularly its emphasis on the interdependence of political commitment, stakeholder coordination, regulatory alignment, capacity building, and financing integration. Across countries, sustained progress was most evident where multiple dimensions reinforced one another, while gaps in even a single area constrained broader institutionalization efforts. Embedding MCCD training within health systems therefore depends not on any single intervention, but on the alignment of governance, regulatory, technical, and financing structures over time. The strategies discussed below should be viewed as connected factors to be prioritized according to health system contexts and available resources, not a fixed sequence.

### Engage stakeholders across sectors

Governments play a central role in institutionalizing MCCD by integrating it into national health strategies, allocating budgetary support, and establishing coordinating mechanisms within health authorities. Because MCCD spans multiple sectors, its implementation also requires collaboration across health, education, and regulatory institutions. However, sustaining this coordination can be challenging in practice. Experiences from another study in Uganda, for example, showed that overlapping mandates, fragmented coordination systems, and competing institutional priorities limited cohesive action even in the presence of formal health legislation [27]. More broadly, findings from this study and the implementation literature suggest that fragmented health systems, unclear accountability, and frequent staff turnover can weaken multi-sector collaboration over time, particularly after external funding ends [5]. These findings highlight that involving multiple stakeholders alone is not sufficient; effective coordination also depends on clear governance structures, aligned responsibilities, and formal mechanisms for collaboration [28,29].

### Establish a clear legislative framework

Clear legal frameworks can support the institutionalization of MCCD training by defining responsibilities, establishing accountability, and embedding training requirements within health and civil registration systems. Legislation may also help formalize the inclusion of MCCD training within medical education, licensure, and accreditation processes, while providing regulatory bodies with the authority to oversee implementation. However, legislation alone does not guarantee sustained change. In many low- and middle-income country settings, broader system priorities such as CRVS strengthening compete with more immediate political and financial priorities, including disease control and maternal and child health programs [30]. In Colombia and Bangladesh, ministerial political support alone was insufficient; without binding legislative requirements, competing institutional interests and faculty autonomy limited progress. These findings suggest that legislative reform efforts must extend beyond health ministries and engage medical education regulators, professional councils, and ministries of finance and education, each of which influences different aspects of implementation, financing, and long-term sustainability.

### Conduct a national assessment of medical certification of cause of death quality

A national assessment of MCCD quality identifies current practices, pinpoints common errors in cause-of-death reporting, and reviews how ICD coding is applied. In several study countries, international partners supported this work by providing standardized tools for systematic assessments. Where resources are limited, rapid assessment approaches using existing administrative data or targeted clinical audit methods can provide sufficient baseline evidence to motivate policy action without requiring large-scale surveys [8].

### Ensure integration with continuous professional development

Evidence has found that mandatory CPD requirements are a strong motivational factor for completion and demonstrably improve practitioners’ knowledge and clinical behavior [15,16]. This suggests that explicitly linking CRVS training to licensure renewal, rather than leaving participation voluntary, is a more reliable pathway to sustained compliance across health workforce cadres. This reflects a broader pattern in health professional education, where administrative tasks are often given lower priority unless supported by formal regulatory requirements [14]. Limited time, heavy clinical workloads, unreliable internet access, and low IT literacy, all affect participation and engagement in completing CPD requirements among practitioners [14]. CPD activities therefore need to be practical and accessible, with formats that fit existing workforce realities and are feasible in both urban and rural settings. In countries where formal CPD systems are still limited, strengthening the broader CPD infrastructure may be an important first step before introducing MCCD-specific requirements.

### Integration of MCCD training

Effective rollout of MCCD training requires approaches that are adapted to different governance structures, infrastructure levels, language needs, and available resources. In decentralized systems, expansion often depends on coordination between national and regional authorities, while more centralized systems may allow for faster and more standardized implementation. Across the countries studied, TOT models emerged as an important strategy for scaling training beyond what could be achieved through centrally delivered programs alone. Countries including Tanzania, Rwanda, Sri Lanka, Bangladesh, and Ethiopia used TOT approaches to expand training to regional and facility levels more efficiently and at lower cost.

Previous studies have also shown that TOT models can be effective in resource-constrained settings, particularly where geographic distance, workforce shortages, and language diversity make centralized training difficult. However, the quality of cascade training depends heavily on the preparation and ongoing support of trainers. In several countries in this study, master trainers were selected from teaching hospitals and referral facilities to maximize downstream reach. At the same time, some settings faced challenges maintaining training quality across multiple levels of rollout, especially where supervision and refresher training were limited.

Regional implementation also raises important equity considerations. Translating materials into multiple local languages, organizing regional training sessions, and releasing clinicians from service duties all require significant time and resources, particularly in rural and under-resourced areas. These findings highlight the importance of incorporating equity considerations into national rollout planning from the outset, rather than addressing them later in the implementation process, as observed in this study’s Ethiopia and Bangladesh experiences.

### Ensure long-term funding and sustainability

External funding often plays an important role in initiating MCCD training programs and supporting curriculum development, digital platforms, and training materials. However, long-term sustainability depends on a gradual transition to domestic financing and integration within national health systems. Across many low- and middle-income countries, securing sustained funding for system-wide priorities such as CRVS and MCCD training remains challenging, particularly when health financing is heavily directed toward disease-specific programs and other immediate health priorities [30,31]. As external funding declines, programs that are not incorporated into routine government budgets often struggle to continue.

The experiences across countries in this study suggest that sustainable institutionalization requires more than short-term project funding. Embedding MCCD training within existing professional development, licensure, and workforce training systems can help reduce dependence on standalone funding streams while strengthening long-term accountability. At the same time, financing decisions are shaped by political priorities, competing resource demands, and perceptions of value within the health system. Advocacy efforts may therefore be more effective when MCCD is framed not as an administrative reporting requirement, but as part of a country’s broader health information infrastructure that supports planning, priority setting, and more accurate public health decision-making [5].

### Study limitations and recommendations for future research

This study has limitations that should be considered when interpreting the findings. First, reliance on self-reported data from key informants may introduce bias. The perspectives of informants captured may not fully reflect the experiences of all actors involved in MCCD training implementation. Second, as noted in the Methods section, members of the research team were involved in supporting MCCD training implementation activities in the countries studied. Although steps were taken to support triangulation and reflexive analysis, this positionality may have influenced interpretation of findings. Finally, the long-term sustainability of donor-supported institutionalization efforts remains an open question that warrants a dedicated follow-up study.

## Conclusion

Accurate cause-of-death data are essential for effective public health policy, and institutionalizing MCCD training is a critical step toward achieving it. Evidence from seven countries across three world regions shows that sustainable integration is possible when supported by strong political will, clear regulation, and coordinated stakeholder engagement. Training alone does not guarantee quality but embedding it within national education and health systems creates the foundation for consistent, reliable certification.

Our findings highlight several success factors. Ministry leadership and early government commitment drove progress where MCCD was treated as a national priority. Integration into both pre-service curricula and in-service CPD built a pipeline of new graduates while addressing gaps among practicing clinicians. E-learning platforms expanded reach, but success depended on infrastructure and user uptake. Linking training to incentives, particularly CPD credits or license renewal, enhances compliance.

Based on these lessons, we recommend: (1) mandating MCCD training through legal and regulatory frameworks; (2) embedding training in medical curricula and CPD systems; (3) scaling user-friendly e-learning platforms alongside in-person workshops; (4) linking completion to licensure renewal and professional incentives; and (5) embedding costs within national budgets to reduce donor dependency.

Institutionalization is not a uniform process; it requires adaptation to national contexts. Yet across diverse settings, common enabling conditions emerge: government leadership, cross-sector coordination, regulatory alignment, and sustainable financing. Countries that embed these elements can strengthen the accuracy of mortality data and strengthen evidence-based health planning and accountability.

## Supplementary Material

COREQ Checklist MCCD STUDY_March 2026.pdf

Supplement A_July 5_ZGHA20260155.docx

## Data Availability

Data supporting the findings of this study are available from the corresponding author upon reasonable request. Due to the qualitative nature of the research and ethical considerations, interview materials are not publicly available.

## Abbreviations

COD: Causes of death
CPD: Continuing professional development
CRVS: Civil Registration and Vital Statistics
ICD: International Classification of Disease and Related Health Problems
MCCD: Medical certification of cause of death
TOT: Training of trainers
