# Supplementary material for: Institutionalizing medical certification of cause of death training: experiences and insights from seven countries
Source: Glob Health Action. 2026 Sep 23;19(1):2732793. doi: 10.1080/16549716.2026.2732793 (PMC13618090; doi:10.1080/16549716.2026.2732793)
Supplement: Supplementary file 1 [file ZGHA_A_2732793_SM5980.pdf]

Supplementary File: Consolidated criteria for reporting qualitative studies (COREQ): 32-item checklist

STUDY/ARTICLE: Institutionalizing Medical Certification of Cause of Death Training: Experiences and Insights from Seven Countries

Item No	Guide Questions/Description	Reported on Page #
**Domain 1: Research team and reflexivity**		
**Personal Characteristics**		
1. Interviewer/ facilitator	Researchers all have experience in global health systems strengthening and CRVS initiatives. Interviews were conducted by the first author RK.	These interviewer characteristics were not explicitly reported in the manuscript because the article focused primarily on policy processes and implementation experiences rather than interviewer reflexivity.
2. Credentials	Researchers include public health specialists and CRVS technical experts with experience in mortality data systems and global health programs. RK has a PhD.	
3. Occupation	Researchers were affiliated with Vital Strategies, Ministries of Health, and independent consulting roles in global public health.	
4. Gender	RK is female.	
5. Experience and training	RK has academic and professional experience in global health initiatives and studies.	
**Relationship with participants**		
6. Relationship established	In most cases, the interviewer had an existing professional relationship with country stakeholders through the Data for Health Initiative.	8
7. Participant knowledge of the interviewer	Participants were aware of the interviewer’s professional role and interest in documenting country experiences related to institutionalizing medical certification of cause of death training.	8
8. Interviewer characteristics	The interviewer had prior experience in global health and civil registration and vital statistics work, which informed familiarity with the topic and context.	8
**Domain 2: study design**		
**Theoretical framework**		

Item No	Guide Questions/Description	Reported on Page #
9. Methodological orientation and Theory	This study employed a qualitative approach to exploring contextual processes, stakeholder experiences, and institutional dynamics that are not adequately captured through quantitative methods.	8
**Participant selection**		
10. Sampling	Countries and participants were purposively selected from those participating in the Bloomberg Philanthropies Data for Health Initiative.	8
11. Method of approach	Country teams were contacted by email with a study concept note.	8
12. Sample size	Seven countries that responded affirmatively to one of the screening questions were included in the study: Bangladesh, Colombia, Ethiopia, Kenya, Rwanda, Sri Lanka and Tanzania.	9
13. Non-participation Setting	Not relevant, as eligibility was determined based on the country teams' responses to screening questions.	N/A
14. Setting of data collection	Virtual semi-structured interviews conducted online.	9
15. Presence of nonparticipants	Non-participants were not present.	N/A
16. Description of sample	Participants included ministry of health officials, health regulatory staff, university deans, and representatives from international organizations involved in MCCD training implementation.	9
**Data collection**		
17. Interview guide	Semi-structured interview guide used (Supplement A).	Supplement A
18. Repeat interviews	No repeat interviews were conducted.	N/A
19. Audio/visual recording	Yes, the audio was recorded for transcription ease later, but only but only with prior written approval from interviewees via email.	9
20. Field notes	Field notes were taken during and immediately after the interviews to support analysis.	N/A
21. Duration	Not reported as it was not relevant for study context, but interviews generally lasted 2 hours with each country.	Not Reported

Item No	Guide Questions/Description	Reported on Page #
22. Data saturation	No, as it was a fixed sample size and not relevant in this context.	N/A
23. Transcripts returned	Yes, interview summaries were shared with country partners for validation.	10
**Domain 3: analysis and findings**		
**Data analysis**		
24. Number of data coders	RK coded the data with collaboration and active feedback from 2 other co-authors.	Not Reported
25. Description of the coding tree	N/A, as themes were developed through iterative thematic analysis rather than a formal coding tree.	N/A
26. Derivation of themes	Themes were derived iteratively from the data, as presented in the results section.	10-20
27. Software	No software used.	N/A
28. Participant checking	Yes, interview summaries and results were shared with country partners for feedback.	10
**Reporting**		
29. Quotations presented	No individual quotations were presented; findings were synthesized and reported at country level to protect anonymity and emphasize cross-country patterns.	N/A
30. Data and findings consistent	Yes, interview summaries and results were shared with country partners to check for consistency between the data presented and the findings.	10
31. Clarity of major themes	Major themes include government commitment, stakeholder engagement, partnerships, training integration, and e-learning platforms.	10-20
32. Clarity of minor themes	Subthemes and contextual examples from countries are described throughout the results and discussion sections.	10-20

Developed from:

Tong A, Sainsbury P, Craig J. Consolidated criteria for reporting qualitative research (COREQ): a 32-item checklist for interviews and focus groups. International Journal for Quality in Health Care. 2007. Volume 19, Number 6: pp. 349 – 357
