# Supplementary material for: Institutionalizing medical certification of cause of death training: experiences and insights from seven countries
Source: Glob Health Action. 2026 Sep 23;19(1):2732793. doi: 10.1080/16549716.2026.2732793 (PMC13618090; doi:10.1080/16549716.2026.2732793)
Supplement: Supplementary file 2 [file ZGHA_A_2732793_SM5979.docx]

**Institutionalizing Medical Certification of Cause of Death Training: Experiences and Insights from Seven Countries**

**Supplement A**
*Guiding Discussion Questions: Multi-Country Experiences of Institutionalizing MCCD Training*

# **Background**

- *Key stakeholders* and players inside and outside the government that, have played a role and/or continue to play a role in advocating for the institutionalization of MCCD?
- Existing documents – what is the *primary legislation* or circular granting mandate to institutionalizing MCCD trainings?
- Have all *legislation processes* required to ensure MCCD trainings are delivered and financed through policy frameworks been completed? If not, are they on track to be completed? What is the expected timeline?
- Were there *previous efforts to institutionalize MCCD* that failed in the past? What worked or did not work during this last attempt?
- Was there an *advocacy phase* with stakeholders such as Medical Associations prior to beginning any formal process around institutionalizing training?
  - *Engagement with relevant authorities responsible for the pre-service curriculum (e.g. ministry of education, deans of medical schools, councils of medical professions).*
- Who have been the *champions of MCCD* progress and activity implementation in your country?

**Incorporating MCCD training into the relevant pre-service curricula**

- Was there a *formal process* to adjust the medical education curriculum? What were the steps and how did this conversation begin? Are there still any pending steps? Yes, MCCOD curriculum was adopted as part of health work force development
- Was the curriculum for the training curricula developed in accordance with *local rules and guidelines, and international best practices*? Which body reviewed that this was adhered to? Is there official documentation of this?
- Who was included in the *curriculum drafting committee*?
- Does the *pre-service curriculum* comprehensively address and cover both clinical and public health aspects of MCCD?
  - *Clinical aspects include: understanding plausible sequences of events leading to death; understanding underlying vs. immediate cause of death)*
  - *Public health aspects include understanding the importance of MCCD data for public health decision-making.*
- Does the MCCD training reflect *clinical training competencies* across various specialties (for example, surgery, medicine, pediatrics, neonatology, oncology, forensics, obstetrics, and primary care)?
- Is an *e-learning platform* and online courses available to medical students? If so, who developed this platform and what was this process like?
  - *The mode of teaching for the pre-service curriculum should be considered carefully and allow for interactive sessions and opportunities for the trainees to discuss and deliberate on example cases.*
- At which stage is this *pre-service training* included into the medical training curricula? (Are trainees at this stage able to understand complex pathophysiology, and are exposed to real cases that enforce the training?)

# **Inclusion of MCCD certification training in continuous medical education programs**

- Was there already any *existing continuous medical education curriculum* or local equivalent that covered MCCD? If so, to what extent?
- Was there a *formal process* to include training on MCCD into the continuous medical education curriculum? What were the steps and how did this conversation begin? Are there still any pending steps?
  - *Were authorities responsible for continuous medical education and accreditation engaged in this process from the beginning? In what way or how?*
  - *Was the training developed in accordance with local rules and guidelines for training curricula? Which body reviewed that this was adhered to? Is there official documentation of this?*
- Who was included in the *training drafting committee*?
- Is this training a required course for *licensure renewal*?
- Have processes been followed for the *accreditation of training modules* with credit points in continuous medical education programs?
- How frequent is *re-training* recommended (i.e. annually or biannually)? Which agency or authority decided this?
- Does the training comprehensively address and cover both *clinical and public health aspects*

of MCCD?

- *Clinical aspects include: understanding plausible sequences of events leading to death; understanding underlying vs. immediate cause of death)*
- *Public health aspects include understanding the importance of MCCD data for public health decision-making.*
- Are an *e-learning platform and/or online courses* available to doctors? If so, who developed this platform and what was this process like?
  - *The mode of teaching for the continuous medical education training should be considered carefully and allow for interactive sessions and opportunities for the trainees to discuss and deliberate on example cases.*
- Are *sessions interactive* and include opportunities for the trainees to discuss extensively using examples and real cases?
  - *The automated ICD mortality coding software Iris, can be considered as an interactive training tool for online training programs.*
- If *face-to-face training* is to be used for continuous medical education, is there a model of training using master trainers? Has training been approved formally by all relevant stakeholders?

# **Monitoring and Evaluation**

- Have any *assessment tools* been developed to be used before and after training on MCCDs? For example, pre and post tests?
- How will the institutionalization be *evaluated for impact*? If not, then what are your opinions on how this might be done?
- In your opinion, what have been the most important *barriers to, and facilitators for* institutionalization of MCCD training? What are the political factors and context which have led to the institutionalization of MCCD training?
- Are there *mechanisms of accountability* in place to monitor that MCCD training is being implemented correctly and consistently?
- In your opinion, what are the *political factors* which will be important to the *ongoing success* of MCCD training?
